# Psychomotor Predictive Processing

**DOI:** 10.3390/e23070806

**Published:** 2021-06-24

**Authors:** Stephen Fox

**Affiliations:** VTT Technical Research Centre of Finland, FI-02150 Espoo, Finland; stephen.fox@vtt.fi; Tel.: +358-40-747-8801

**Keywords:** active inference, critical realism, embodiment, experience, human–robot, pain, hierarchical predictive processing, predictive global neuronal workspace, psychomotor, Society 5.0

## Abstract

Psychomotor experience can be based on what people predict they will experience, rather than on sensory inputs. It has been argued that disconnects between human experience and sensory inputs can be addressed better through further development of predictive processing theory. In this paper, the scope of predictive processing theory is extended through three developments. First, by going beyond previous studies that have encompassed embodied cognition but have not addressed some fundamental aspects of psychomotor functioning. Second, by proposing a scientific basis for explaining predictive processing that spans objective neuroscience and subjective experience. Third, by providing an explanation of predictive processing that can be incorporated into the planning and operation of systems involving robots and other new technologies. This is necessary because such systems are becoming increasingly common and move us farther away from the hunter-gatherer lifestyles within which our psychomotor functioning evolved. For example, beliefs that workplace robots are threatening can generate anxiety, while wearing hardware, such as augmented reality headsets and exoskeletons, can impede the natural functioning of psychomotor systems. The primary contribution of the paper is the introduction of a new formulation of hierarchical predictive processing that is focused on psychomotor functioning.

## 1. Introduction

Within predictive processing, perception is a Bayesian process that involves updating prior beliefs into posterior beliefs in order to reduce prediction errors, in other words, to reduce differences between what we expect to experience and what we do experience. Empirical research by others has provided support for predictive processing. For example, interactions between predictions, expectations, sensory inputs, and attention have been found in research using functional magnetic resonance imaging [1]. Other empirical studies deploying neuroimaging have observed neurology for prediction errors [2], while separate empirical research using magneto-encephalography has found interactions between predictions and prediction errors [3]. In addition, empirical research by others using magneto-encephalography has examined disruptions to hierarchical predictive processing caused by sleep [4].

Prediction errors can be minimized by changing our expectations and/or changing our actions, including changing what we pay attention to and/or changing what we do. Notably, changes to reduce prediction errors may not improve wellbeing. For example, chronic ill health can be influenced more by changes to expectations and attention than by sensory evidence [5,6]. Apropos, findings from empirical research by others indicate that pain experience can be predicted in Bayesian terms [7]. Furthermore, findings from empirical research by others have indicated that symptoms can reflect the relative predominance of either prior beliefs or sensory inputs with predictive processing being influenced by personal characteristics [8]. 

In this paper, predictive processing was addressed from the point-of-view of planning and operating systems involving humans interacting with robots and wearing technologies such as augmented reality headsets and exoskeletons. This is necessary because such systems are becoming increasingly common and move us farther away from the hunter-gatherer lifestyles within which our psychomotor functioning evolved [9]. For example, beliefs that workplace robots are threatening can generate anxiety and fear, while the use of hardware, such as augmented reality headsets and wearable exoskeletons, can impede the natural functioning of psychomotor systems [10].

In particular, the purpose of this paper was to expand the scope of predictive processing theory in three ways. First, by going beyond previous studies that have encompassed embodied cognition but have not addressed some fundamental aspects of psychomotor functioning. Second, by proposing a scientific basis for explaining predictive processing that spans objective neuroscience and subjective experience. Third, by providing an explanation of predictive processing that can be incorporated into the planning and operation of systems involving robots and other new technologies. Throughout the remainder of this paper, such systems are referred to as human–robot systems.

To fulfil this purpose, the paper proceeds in seven further sections. In Section 2, the need to incorporate psychomotor predictive processing in the planning and operation of human–robot systems is explained. In Section 3, it is explained why the critical realist philosophy of science provides a better basis for framing psychomotor predictive processing than either the positivist or anti-positivist philosophies of science. In Section 4, it is explained that predictive global neuronal workspace (PGNW) [11] is the theory that is most compatible with critical realist framing of psychomotor predictive processing. PGNW combines predictive processing with the global neuronal workspace explanation of consciousness, the support for which can be found in more than a decade of empirical research by others using, for example, electroencephalography [12]. In Section 5, a critical realist framing is described with two examples. In Section 6, a critical realist framing of PGNW is related to psychomotor experience. In Section 7, a new formulation of hierarchical predictive processing is proposed that is focused on psychomotor functioning. In addition, challenges and opportunities are discussed for including the consideration of hierarchical psychomotor predictive processing in the planning and operation of human–robot systems. In Section 8, principal contributions are stated and directions for further research are proposed. Overall, this paper concerns theory building, principally, by bringing together the critical realist philosophy of science and PGNW in a new formulation of hierarchical predictive processing that is focused on psychomotor functioning. Although this theory building does not include new primary data from experiments, extensive reference is made to empirical research by others. In addition, the limitations of previous research and the need for further research are discussed in Section 6 and Section 8, respectively.

## 2. Need for Predictive Processing Formulation Focused on Psychomotor Functioning

New technologies bring an increasing variety and frequency of novel sensations that are far from those that human psychomotor functioning evolved to deal with [9,13]. For example, exoskeletons are mechanical frameworks that humans can wear to increase their strength and endurance beyond our evolved psychomotor limits. However, the wearing of exoskeletons restricts the range of human motion, such as three-dimensional rotational movements that are typically involved in lifting. At the same time, the wearing of exoskeletons introduces multiple new and unpredictable loads to the musculoskeletal systems. Hence, the wearing of exoskeletons can have negative effects on fascia system functioning. The fascia system comprises bands and sheets of connective tissue beneath the skin. It attaches, stabilizes, encloses, and separates muscles. Importantly, the fascia system is our largest sensory organ for interoception, nociception, and proprioception. Hence, exoskeletons can involve immediately apparent novel sensations, for example, from lifting while wearing a mechanical framework. Then, over time, wearing exoskeletons could contribute to unpredictable novel sensations through negative unintended changes to interoception, nociception, and proprioception. Here, it is important to note that wearable exoskeletons can be used in conjunction with other new technologies that introduce further sources of novel sensations, such as augmented reality headsets and mobile robotics [10,14]. 

Moreover, although fabricating new technologies involves more extraction of finite natural resources from the lithosphere and more disruption to the biosphere [15], the deployment of robots, etc. is increasing in many sectors [16,17]. Yet, the anticipation of widespread full automation is now being revised to the planning and operation of human–robot systems in what has been described as Society 5.0 [18]. This term refers to the fifth stage of technology-enabled development since the beginning of the industrial revolution, in which the need for technologies to complement and enhance human capabilities is emphasized [19]. In particular, rather than aiming for full automation, it is recognized that the so-called human touch is important in many endeavors [20], including in production work [21] and in healthcare [22].

This technology-enabled movement toward so-called Society 5.0 encompasses the consideration of human beliefs, including ethics [23]. Human ethical behavior is often a psychomotor phenomenon within which most people will typically adhere to sociocultural ethical practice unless overloaded by stress [24,25], for example, from resource depletion and time pressure [26,27]. Accordingly, from an optimistic perspective, human–robot systems have the potential to facilitate ethical behavior if robots reduce human stress from workload and time pressure. On the other hand, the introduction of robots can cause human stress to increase. For example, as well as anxieties about robots taking jobs [28]; there are also anxieties about robots developing dangerous superintelligence; about robots harboring malicious intrinsic motivations and about robots enacting unfavorable intentions [29,30,31,32]. Notably, human anxiety about perceived threats can contribute to humans underestimating their proximity [33] and overestimating the pain that they could cause [34]. Moreover, psychological stress from anxiety can contribute to people becoming accident-prone [35].

Hence, human–robot systems need to be planned and operated to minimize the potential for increasing human stress. Here, it is important to note that different people have different propensities for experiences of anxiety and related pain [36,37,38]. Apropos, the planning and operation of human–robot systems need to take into account psychomotor differences in a wide variety of settings that could contribute to unintended consequences, including increased human stress, anxiety, and pain. This is of the utmost importance because short-term stress, anxiety, and pain can contribute to long-term disorders of consciousness, such as the experience of chronic symptoms without pathophysiological disruption. In other words, chronic ill health that is far more extensive than potential initial causes, i.e., chronic medically unexplained symptoms [5,6,39,40]. Accordingly, it is important to include consideration of predictive processing in the planning and operation of human–robot systems. In particular, hierarchical predictive processing provides a physics of life perspective for psychomotor experience, which encompasses novel sensory inputs being followed by the development of negative expectations, and then the avoidance of movement and other sources of sensory inputs that are expected to be negative [41,42]. 

The consideration of predictive processing should be carried out with methods that are consistent with the preference of science and practice for parsimony and simplicity [43,44,45,46,47]. Accordingly, methods should be informed by a scientific theory that provides a simple predictive framework for psychomotor experience. This should be a scientific theory that is focused upon the embodied action that is inherent in psychomotor functioning. Furthermore, it should be compatible with the parsimonious simple methods used in engineering design and operations management. Typically, this involves formats such as tables within methodologies that provide structured guidance for dealing with otherwise challenging issues. For example, design methodologies can include tables for function analyses, quality function deployment, and evaluating alternatives [48].

## 3. Critical Realist Philosophy of Science

There is debate about different levels and types of consciousness [49,50,51,52,53,54]. These can be analyzed in terms of, for example, physical-neurobiological, functional, informational-computational, representationalist, and phenomenological [55]. Underlying debate about levels and types of consciousness is the difficulty of bringing together opposing philosophies of science: positivism and anti-positivism, for example, positivist neuroscience data and anti-positivist interpretivist descriptions. Critical realism is a philosophy of science that bridges positivism and anti-positivism [56], and it is used in the framing of psychomotor experience because of its advantages compared to positivism and anti-positivism [57,58]. In particular, unlike positivism’s general laws in flat conjunctions of cause and effect, critical realism posits a three-layered stratification of causation encompassing the why, how, and what of experience. Furthermore, unlike anti-positivism within which it is claimed that explanations can provide only subjective impressions of unique human experiences, critical realism provides why-how-what explanations of causation that are generalizable within particular enabling contexts [59,60,61].

Critical realist context-dependent causation (i.e., why) involves generative causal mechanisms comprising tendencies and powers [62,63]. Tendencies are potentials that are typical to a category. For example, many people can be anxious occasionally but people within the neurotic personality type category possess the tendency to suffer anxiety more often [64]. Powers are potentials to do specific things but not others. For example, the human body has a wide, but not limitless, range of movement [65].

Critical realist reality is an open system where causation can be generalizable, but only within contexts that provide conditions for enabling causation. For example, psychomotor rehabilitation treatment can be carried out within different organizational structures, such as building arrangements, and personnel roles, and within different organizational cultures, such as dress code and communication style. However, if organizational structure and organizational culture for psychomotor rehabilitation are the same for all rehabilitation treatments, such as physiotherapy, then different psychomotor experiences from the same treatment are likely to arise from different generative causal mechanisms.

Notably, critical realism provides a generalizable causal framing for the same actions in the same context, such as the same environmental niche, leading to different experiences. This is different to positivistic laws of causation. It is also different to the anti-positive view that an action can lead to diverse outcomes that are too individually subjective to be generalized. However, unlike anti-positivism, the critical realist explanation is not antagonistic toward those starting from a positivistic perspective. This is because, in common with positivism, critical realism holds that it is possible to construct knowledge that, to some extent, represents or mirrors reality as it objectively exists. At the same time, unlike positivism, the critical realism explanation is not antagonistic toward those starting from an anti-positivistic perspective. This is because, in common with anti-positivism, critical realism holds that there are no universal mechanisms of causation in human endeavors. Hence, critical realism can bridge otherwise opposing perspectives [66].

## 4. Predictive Global Neuronal Workspace Theory (PGNW)

There are many theories of consciousness, each which has its own strengths and weaknesses [67]. However, the scientific theory of consciousness that is most relevant to psychomotor predictive processing is predictive global neuronal workspace theory (PGNW) [11,68,69].

PGNW has several advantages for including psychomotor generative characteristics in the planning of operation of human–robot systems. In particular, it is has the underlying simplicity of a widely applicable principle: the free-energy principle (FEP). This is a physics of life principle, which formalizes embodied cognition of the autopoietic organization of living things. The FEP formalizes that active systems must occupy a limited repertoire of internal states through minimizing the long-term average of unwanted surprise from external states: i.e., from the world [70,71].

For psychomotor experience, a further advantage is that the corollary of FEP applied within PGNW is active inference by which living things take action in order to survive. Specifically, in this physics of life process theory, living things, including humans, implement generative models in order to survive. This involves humans surviving by taking action to align their internal generative models with external states. Apropos, free energy in FEP can be regarded as the information a person is lacking to align her/his internal generative model with the external state [41]. Hence, if survival depends upon resolving information gaps between the internal generative model and the external state, then free energy in FEP can be regarded as the survival information deficit from which unsustainable unwanted surprise arises. Action to better align the internal generative model with the external state reduces unwanted surprise by reducing the survival information deficit (i.e., by reducing FEP free energy). Better aligning can involve updating the internal generative model based on current sensory sampling of the external state and current actions taken in the external state. Alternatively, updating the internal generative model can be based on the action of changing what sensations are sampled from the external state, and/or can be based on taking new action in the external state to change sensations from the external state. For example, changing sampling can involve becoming hypervigilant to a potential source of pain, and taking new action in the external state to restrict work involving the potential source of pain. Often, actions will be interrelated, and can be referred to as a policy, with new priorities for sensory sampling and/or new priorities for actions being a policy change. Overall, humans survive by reducing prediction errors amidst the complexity of the environmental niches in which they survive [72]. Active inference is relevant to psychomotor experience in many different practical settings [73].

Another distinction of PGNW is that it has a parsimonious structure, which is very suitable for tabular representations. Specifically, PGNW comprises three main constructs: the consistency of top-down prior expectations, strength of bottom-up sensory signals, and endogenous attention-based modulation. These are constructs that have been found to be important in separate empirical research by others, for example, in relation to the experience of pain. Research deploying neuroimaging has found that expectations shape individuals’ experiences of pain [74], and that attention affects which sensory events are selected [75]. Notably, as explained in more detail in subsequent sections with further references, findings from other empirical research also indicate that constructs in PGNW can be influenced by characteristics such as personality type [76,77].

Importantly, PGNW draws upon one of the most empirically well-supported models of consciousness: global neuronal workspace [12]. Another advantage is that within critical realism, causation is generative [62,63] and generative models are at the core of PGNW. Notably, through its predictive potential, PGNW has potential to contribute to addressing the psychomotor experience of symptoms without pathophysiological disruption [5,6]: for example, negative changes in psychomotor functioning that are much larger and more long-term than can be explained by a potential cause, such as a minor injury.

## 5. Critical Realist Framing of Psychomotor Experience

Embodied cognition involves mind–body behaviors that are not always brain-centric [78,79], and can be described as complex psychomotor interactions [80,81]. For example, our largest sensory organ for interoception, nociception, and proprioception is the highly innervated fascial system [82,83]. The fascia system can become densified around our physiological asymmetries, such as one leg being slightly shorter than the other, during repetitive movement patterns in modern lifestyles [84,85]. This can shape how we walk (i.e., our gait), which can affect what we remember [86] and influence our personalities [87]. This is an example of mind–body behaviors involving complex psychomotor interactions, rather than being wholly brain-based. Apropos, the same pain stimuli can be followed by different people experiencing different pain [88,89].

As summarized in Table 1, nonconscious combinations of personality type, hardiness level, fascia densification, and body memory can entail generative causal mechanisms for a low probability of conscious pain experience or high probability of conscious pain experience. This critical realist generative perspective of pain causation is consistent with pain matrix theory [90,91].

With regard to tendencies, personality type can affect pain experience. For example, higher neuroticism and lower openness are associated with persistent pain [92]. Alongside personality type, hardiness is an attribute that allows some individuals to stay healthier under stress than others [93,94]. Information for the definition of tendencies (what) can be obtained through other established techniques such as personality type tests and hardiness measure scales [95]. Information for the definition of current experience (what) can be obtained through other established techniques such as self-reporting pain scales, physical examination, and imaging studies.

With regard to powers, the highly innervated fascial system comprises tissues that connect bones with muscles to enable dynamic functioning [96,97]. However, dynamic functioning depends upon fascia being able to move fully and smoothly. Densification can restrict fascial functioning by restricting sliding between fascial tissue interfaces. Densification can result from over-repetition of a narrow range of movements [98]. Importantly, expectations about future pain can be based on body memory of past pain [99,100,101]. Information for the definition of powers can be obtained through established techniques such as physiological function tests [102] and pain memory questionnaires [103].

As summarized in Table 1, a nonconscious generative causal mechanism for a low probability of pain experience would be, for example, a person not being prone to pain anxiety, having a high hardiness, having one minor fascia densification, and body memory of one fast pain recovery. For such a person, one surprise pain from movement during a routine narrow range of movements can be experienced due to one minor densification in the fascial system. Such densification could be reversed through one physical therapy involving fascia manipulation and small rehabilitation exercise to restore full smooth fascia functioning [104,105]. This could be followed by a reduced likelihood of further pain as the range of movement is increased through movement practice and the perception is improved through reduced densification of the fascia system [106].

By contrast, a generative causal mechanism for a high probability of pain experience would be, for example, a person being prone to pain anxiety, having low hardiness, having widespread fascia densification, and having body memory of many persistent pain experiences. Consider, for example, a prescribed rehabilitation exercise for such a person. Rehabilitation exercise can be followed by slightly increased pain initially [107]. However, this can have the unintended negative consequence of reducing the range of movement as the person tries to avoid pain. This rehabilitation exercise outcome can precipitate learned helplessness and descend into chronic pain conditions with widespread complex psychomotor symptoms [108,109,110,111,112]. The potential for such negative unintended consequences may be increased through stress caused by interacting with robotics and the wearing of hardware such as augmented reality headsets and exoskeletons [10,14]. Accordingly, it is appropriate to include consideration of generative causal mechanisms, as illustrated in Table 1, during the planning and operation of human–robot systems.

## 6. Relating PGNW to Critical Realist Framing of Psychomotor Experience

### 6.1. Overview

As summarized in Table 2, PGNW can be related to an expanded version of the examples above. Apropos, a human production operative who works with mobile robotics while wearing hardware, such as exoskeletons and headsets, may develop a consistent top-down prior expectation of ankle pain from what was initially a surprise ankle pain early in a working day. This could have arisen from wearing an exoskeleton having introduced unpredictable loads to the production operative’s musculoskeletal system while restricting three-dimensional rotational movements that are typically involved in lifting. Toward the end of the day, there may be very strong bottom-up sensory signals because the operative has to do some heavy lifting. If the operative pays attention to the ankle, then it is likely that the operative will be conscious of ankle pain. However, as the end of the working day nears, the operative’s attention to ankle pain can be modulated by attention being focused on leaving work on time so as to be able to keep a social appointment. As a consequence, the operative may not be conscious of ankle pain at the final stages of the day despite there being several hours of consistent prior evidence to support ankle pain expectation and despite strong physical causation for increased ankle pain during heavy lifting [113,114]. Whether conscious or nonconscious of ankle pain at the end of the working day, the operative may subsequently undergo some minor ankle fascia manipulation therapy and begin one ankle rehabilitation exercise to widen the range of ankle movement. After a few days, the ankle may be pain-free and the operative may soon forget about having had ankle pain.

However, the wearing of exoskeletons could have negative effects on fascia system functioning, such as negative unintended changes to interoception, nociception, and proprioception [9]. Apropos, the next day, the operative’s anxiety about ankle injury could lead the operative to becoming hypervigilant to any bottom-up sensory signal related to the ankle. This can involve two attentional processes of hypervigilance: detection of threatening stimuli and difficulty in disengaging attention from threatening stimuli. In particular, operatives with high pain-related anxiety are more likely to orient their attention toward a pain-related threat and have difficulty in disengaging from the threat [115]. Treatment may not end hypervigilance. Rather, treatment can be followed by fear of re-injury and prevent the return to work [116]. At worst, they can descend into chronic fear of pain from movement (i.e., kinesiophobia) and chronic pain conditions with widespread complex symptoms, such as fibromyalgia [117]. Thus, formulation of the prior expectation of acute ankle-specific pain during one working day can expand into a consistent prior expectation of chronic body-wide pain. Here, it is important to note chronic body-wide pain is prevalent amongst many populations throughout the world [118]. 

### 6.2. Consistency of Top-Down Prior Expectations

During the evolution of our psychomotor systems, humans were hunter-gatherers who could develop consistent prior expectations as they moved on foot through natural environments in order to survive [119]. The change from hunter-gathering to agricultural settlement began the transition away from natural lifestyles [120] and toward a disconnect between psychomotor movement and human survival. This has brought a fundamental change in prior expectations. In particular, hunter-gathers have the prior expectation that survival depends upon moving with the wide repertoire of full-body motions involved in hunting and gathering. By contrast, the majority of humans in 2021 are not involved in the daily psychomotor motions involved in hunting and gathering. Rather, the majority of humans can have the consistent prior expectation that survival will not depend upon undertaking a wide repertoire of daily full-body motions [121]. There is evidence that humans become conscious of the pain that they are expecting to experience [89]. Yet, having expectations of pain from movement alongside no expectation of having to move in order to survive is a fundamentally different combination of expectations compared to when the human psychomotor system evolved. This disconnect between movement and survival can be addressed through psychomotor rehabilitation to increase movement. However, the compositions of prior expectations related to psychomotor pain are very difficult to define and to address [122,123], not least because of the lack of understanding of how body memory functions [124,125]. Meanwhile, the world’s remaining hunter-gatherers continue to have better health than much of the rest of humanity [126].

### 6.3. Strength of Bottom-Up Sensory Signals

If there is a strong pain signal from a strong blow, there can be a close match between the point of contact and location of short-term pain. By contrast, in phenomena such as complex regional pain syndrome (CRPS), there can be widespread pain disproportionate in severity and time to the original source of pain. This can involve extreme pain, swelling, reduced motion range, changes to skin, and changes to bones. CRPS can start in one limb but spread throughout the body [127]. CRPS pain is an example of diffuse pain, which is not fully understood by medical science [128]. In addition to phenomena within which pain signals are not connected to an initial pain source and pain signals being diffuse, pain signals can also be unpredictable [129].

Yet, even a very strong bottom-up psychomotor signal does not necessarily lead to pain. For example, in the zone of a psychomotor flow state [130,131], a boxer may not be conscious of pain from punches. This is epitomized by the words of the famously successful boxer, who went 91 bouts undefeated between 1943 and 1951, Sugar Ray Robinson: “You don’t think. It is all instinct. If you stop to think, you’re gone” [132]. Hence, he was in the zone of flow states long before sports psychology formalized them in scientific research and coaching practice [133]. More generally, repeated strong sensory signals, for example, in contact sports, can contribute to the same pain stimuli involving lower pain [134]. Thus, pain signals are different to audio signals and visual signals, which have been previously addressed in PGNW research, that are directly related to sources, that are specific, that are predictable, and that are not overridden by flow states [68,135].

### 6.4. Endogenous Attention-Based Modulation

With regard to attention and awareness, within PGNW, the more likely a top-down prior expectation is to predict a bottom-up sensory signal (i.e., the higher the prior probability), the more attention will be paid to the prior expectation and the more influence the prior expectation will have on what is experienced. By contrast, the lower the prior probability, the more attention will be paid to the bottom-up sensory signal and any prediction error between prior expectation and what is actually experienced. Thus, the predictive in predictive global neuronal workspace (PGNW) draws upon active inference: a corollary of the free-energy principle (FEP) according to which a self-organizing system will maintain itself by staying within a narrow range of states consistent with its survival. This involves all self-organizing systems, including humans, needing to minimize unwanted surprise from prediction errors [67]. However, the modern disconnect between psychomotor movement and survival leads to disconnects between minimizing unwanted surprise and survival. For example, as discussed above, fear of pain surprise can contribute to persistent extreme attention to pain and minimizing movement to avoid pain, thus acting against the survival imperative for psychomotor movement [136,137].

Within PGNW, conscious awareness should be continuous with an inferential hierarchy involving nonconscious processing. This entails the stability of conscious representations by superordinate representations being synchronized with subordinate representations. For example, conscious superordinate representation of a tool box is synchronized with subordinate representations of the surfaces, edges, and colors of the tool box. In turn, a superordinate model of a tool box can be embedded into scenes, such as a work site, in order to generate predictions about the ways that the tool box will be moved by a fork lift truck. Synchronous subordinate representation is needed to prevent loss of precision in representation of the tool box as it is moved [138]. However, unlike hierarchical PGNW models, psychomotor experience does not necessarily arise from temporally synchronous hierarchies. For example, the fascia system comprises complex matrices with indeterminate shifting boundaries rather than a stable hierarchy [139]. Moreover, psychomotor pain arises through complex interactions between, for example, personality type, hardiness levels, fascia, and body memory, which do not take place in synchronized hierarchies [140]. For example, psychomotor action can take place during nocturnal scratching of dermatitis while sleeping [141].

### 6.5. Expectations, Signals, Attention, and Human–Robot Systems

Human interactions with robots involve top-down prior expectations, the strength of bottom-up sensory signals, and endogenous attention-based modulation. Depending upon the human’s nonconscious generative mechanism, the influence of human–robot interaction can be more likely to have a positive effect on human psychomotor experience or be more likely to have a negative effect on human psychomotor experience. For example, design engineers and computer scientists who develop human–robot systems need to take into account that human top-down expectations can be negative. Moreover, negative expectations can contribute to negative human perception of bottom-up signals and negative human endogenous attention-based modulation, which, in turn, can contribute to negative consequences for psychomotor predictive processing. Furthermore, those who operate and manage human–robot systems need to take into account that stress and anxiety from human–robot interactions have the potential to contribute to long-term disorders of consciousness, for example, the experience of chronic symptoms without pathophysiological disruption [5,6,39,40]. Here, it is important to note that once individuals get “stuck” in chronic symptoms, it can take very long-term interventions to get “unstuck” [41]. Accordingly, the planning and the operation need to take into account different human nonconscious generative mechanisms (critical realist-why) and how these can provide the underlying basis for many activity-specific human generative models (PGNW-how) related to a wide variety of human interactions with robots, and with hardware such as augmented reality headsets and wearable exoskeletons.

## 7. Psychomotor Hierarchical Predictive Processing

### 7.1. Overview

**Figure 1:**
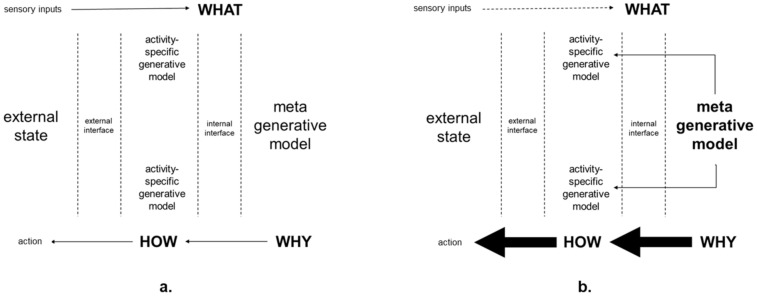
Critical realist generative causal mechanism as a meta-generative model in hierarchical predictive processing: (**a**) in balance with sensory inputs; (**b**) determines attention, expectation, and action irrespective of sensory inputs.

The introduction of critical realist framing for nonconscious generative causation provides a unifying explanatory basis for activity-specific generative models that are focused on reducing prediction errors related to particular activities. For example, a human worker may have particular generative models for getting ready to travel to work, for travelling to work, for doing work, and so on. By contrast, as summarized in Table 1, critical realist generative causal mechanisms are not focused directly on prediction error reduction in particular individual activities. Rather, they can underlie all of a person’s activity-specific generative models. Accordingly, as summarized in Figure 1, they can be considered meta-generative models that are consistent with theoretical constructs such as self-models [79,142,143] and hierarchical predictive processing [41,42,144]. These are meta-generative models of individuals’ own psychomotor lives in the world as the world is experienced by those individuals. In other words, they are subjective autopoietic meta-generative models of world/self. Such meta-generative models can provide an explanatory basis for practitioners, such as design engineers and computer scientists, about why some people can suffer the descent into chronic ill health without pathophysiological disruption. For example, as summarized in Figure 1b, consistently strong top-down prior expectations of pain, which override bottom-up sensory inputs, can come from a generative causal mechanism/meta-generative model comprising a combination of personality type prone to pain anxiety, low hardiness, widespread major fascia densification, and adverse body memories. Accordingly, design engineers and computer scientists should develop human–robot systems to minimize the potential for increasing human stress. In particular, human–robot systems should be developed to maintain balance between the meta-generative model and sensory inputs shown in Figure 1a.

### 7.2. Challenges

Even with a critical realist framing, there are challenges for the application of modeling methods used in previous PGNW studies during the planning and operation of human–robot systems. For example, methodologies for defining personality type, hardiness levels, fascia densification, and body memory can contribute to approximate descriptions with indeterminate boundaries, such as those between one personality type category and another [145]. Moreover, fascia research is at an early stage [146]. Hence, it is as yet unclear exactly how fascia densification affects interoception, nociception, proprioception, and exteroception. In addition, relationships between body memory and fascia are topics of scientific research that are at a very early stage. Accordingly, the influence of fascia densification and body memory on bottom-up sensory signals is uncertain. With regard to attention-based modulation, the fascia system comprises complex matrices, rather than a stable hierarchy, within which the operation of body memory is uncertain. Nonetheless, critical realist PGNW already has potential for incorporation into the planning and operation of human–robot systems.

### 7.3. Opportunities in Planning Human–Robot Systems

**Figure 2:**
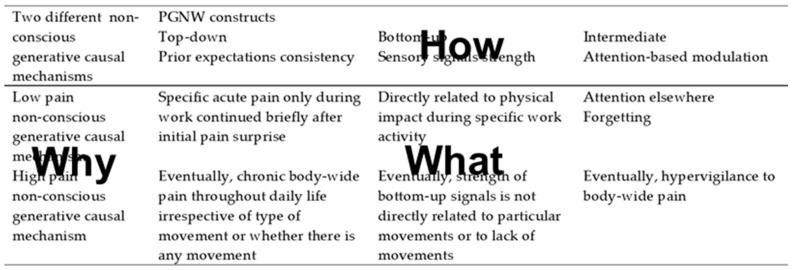
Critical realist PNGW why, how, and what.

With regard to planning, as shown by Table 2 and Figure 2, critical realist PGNW has the characteristic of good scientific theories and of engineering design methods: parsimony [147]. In particular, critical realist PGNW is parsimonious in its definition of main constructs and interactions between them. For example, a main proposition is that consciousness is most likely to occur when there is alignment between consistent top-down prior expectations, strong bottom-up sensory signals, and endogenous attention-based modulation. As summarized in Table 2, this fundamental proposition is applicable to the critical realist framing of psychomotor experience and is a useful starting point for setting-out nuances, divergences, and their consequences. 

For example, within psychomotor experience, as explained above, there is no need for pain signals to be strong in order for there to be strong pain. Rather, hypervigilance to the fear of pain can contribute to descent into debilitating diffuse body-wide pain. Conversely, strong pain signals do not necessarily lead to strong pain. Rather, there are situations where people pay little, if any, attention to strong pain signals, for example, when in the zone of flow states. Moreover, the modern disconnect between psychomotor movement and survival subverts assumptions that minimizing unwanted surprise will facilitate survival. On the contrary, as explained above, minimizing unwanted pain surprise through avoiding movement can contribute to chronic widespread pain and many serious health problems. Accordingly, tabular summaries of critical realist PGNW can be used to highlight these issues in conjunction with established engineering design methods such as failure mode and effects analysis (FMEA) and job design. FMEA provides a systematic process for predicting and preventing problems [148], while job design methods are used to facilitate both workplace performance and worker wellbeing [149].

Here, it is important to note that human participation in human–robot systems is not limited to those who operate the systems. Rather, they can involve human end-users who may already be facing psychomotor challenges, such as elderly residents in care homes that deploy robotics to assist human carers. Thus, critical realist PGNW could be included in service design [150] that takes into account the psychomotor characteristics of human-robot systems’ end-users such as care home residents.

### 7.4. Opportunities in Operating Human–Robot Systems

With regard to the operation of human–robot systems, critical realist PGNW can be useful in neuroscience education for successful psychomotor experiences and for addressing poor psychomotor experiences [151]. This can include using critical realist PGNW as a basis for calibrating expectations [152] by relating possible actions to different nonconscious generative causal mechanisms, for example, as summarized in Table 1 and Table 2. This can better enable explanations to be accepted, for example, by reconciling personal goals with personal characteristics [153]. This can minimize what has been described as the expectation–actuality discrepancy (i.e., prediction error) and thus increase the likelihood of satisfaction [154] through the definition of attainable states [41]. In particular, the definition of attainable states [41] can reduce negative consequences from expectations being set either too high or too low [155].

For explanation by humans, no major additions to the current best practice are required for critical realist PGNW to support neuroscience education for practitioners. The design of critical realist PGNW information and its communication should be based on the best practice [156,157,158] to enable accessibility to the individual and all those who can support the individual in progressing, for example, toward psychomotor pain reduction [153,154]. For explanation by artificial intelligence (AI), critical realist PGNW descriptions are well-suited to the current third phase in the development of AI. Specifically, a third phase that aims to combine the knowledge-guided top-down basis of its first phase with the data-driven bottom-up basis of its second phase [159,160]. This hybrid AI is not monolithic but is open to top-down rules-based software from different vendors and data-driven bottom-up software from other vendors. For example, critical realist PGNW constructs and interactions between them could be maintained in top-down standardized templates in one software, and details of individuals’ characteristics could be bottom-up inputs from many other software packages. Importantly for human accessibility, it may be possible for both top-down rules and bottom-up inputs to be human-readable [161]. This can be important to both operatives and end-users in human–robot systems.

## 8. Conclusions

The purpose of this paper is to expand the scope of predictive processing in three ways. First, by going beyond previous predictive processing studies that have encompassed embodied cognition but have not addressed fundamental aspects of psychomotor functioning [162]. As summarized in Table 1, this has been carried out by providing preliminary analyses of interrelationships between personality type, hardiness levels, fascia system, and body memory. Second, the scope of predictive processing has been expanded by proposing a scientific basis for explaining predictive processing that spans objective neuroscience and subjective experience. This has been carried out by explaining how critical realism addresses the comparative limitations of positivism and anti-positivism, while providing a bridge between them. Furthermore, as summarized in Table 1, it has been explained that critical realism is inherently aligned with predictive processing through its emphasis on the generative nature of causation. Third, the scope of predictive processing has been expanded by providing an explanation of predictive processing that can be incorporated into the planning and operation of systems involving robots and other new technologies. This has been carried out through the practical analysis summarized in Table 2, Figure 1 and Figure 2, which are typical of the tabular formats and simple diagrams that are widely used in engineering design [48].

As summarized in Figure 1, the primary contribution of the paper was the introduction of a new formulation of hierarchical predictive processing, which is focused on psychomotor functioning. This formulation is not in competition with extant theoretical formulations, which have also been based on reviews of previous empirical research and encompass hierarchical predictive processing [41,79,163,164]. Rather, it is complementary to extant theoretical formulations encompassing hierarchical predictive processing, but which are not focused on psychomotor experience. Furthermore, the primary contribution of this paper is relevant to various theoretical formulations concerned with embodied, embedded, enacted, and extended cognition (4E) [165], which involve, but have not addressed, fundamental aspects of psychomotor functioning that are included in this paper. 4E cognition assumes that cognition is shaped by dynamic interactions between the brain, body physical environment, and social environment. Yet, previously, 4E studies in the literature have not encompassed interactions between personality type, hardiness levels, fascia system, and body memory, nor have they provided a structuring of the why, how, and what of psychomotor experience that can inform the planning and operation of human–robot systems. This is important because 4E cognition faces new challenges from interacting with robotics and wearing new technologies such as augmented reality headsets and exoskeletons.

A further important contribution was the outline of challenges for the application of modeling methods used in previous PGNW studies. In Table 3, a summary is provided of directions for further research to advance the understanding of psychomotor predictive processing, and its consideration in the planning and operation of human–robot systems.

Meanwhile, there are already many potential future directions for applying critical realist PGNW. These include furthering predictive processing research into skilful psychomotor performance in general [166] and specific phenomena in skilful psychomotor performance such as kinaesthetic motor imagery [167]. Such research can inform efforts to design and maintain systems that facilitate the balance between top-down expectations and bottom-up sensory inputs, as summarized in Figure 1a. With regard to Figure 1b, future research could also include addressing the psychomotor experience of symptoms without pathophysiological disruption and the psychomotor experience of relief after the administration of placebo treatments [6]. Such research could include how human nonconscious and conscious beliefs about robots and other technologies affect workplace performance and personal health. Furthermore, relating predictions framed in terms of critical realist PGNW to subsequent rich descriptions of what has been experienced [168] can provide wider and deeper insights that can be informative for all scientists and practitioners with interest in predictive processing.

## Figures and Tables

**Table 1:** Examples of non-conscious psychomotor generative causal mechanisms.

Critical Realist Non-Conscious Generative Causal Mechanism		Low Pain Non-Conscious Generative Mechanism	High Pain Non-Conscious Generative Mechanism
Tendencies	Personality type	Not prone to pain anxiety	Prone to pain anxiety
	Hardiness level	High	Low
Powers	Fascia densification	Minor fascia densification	Widespread fascia densification
	Body memories	Fast recovery from local pain	Widespread persistent pain

**Table 2:** Two examples of relating PGNW to critical realist psychomotor experience.

Two Different Non-Conscious Generative Causal Mechanisms	PGNW Constructs		
	Top-Down	Bottom-Up	Intermediate
	Prior Expectations Consistency	Sensory Signals Strength	Attention-Based Modulation
Low pain non-conscious generative causal mechanism	Specific acute pain only during work continued briefly after initial pain	Directly related to physical impact during specific work activity	Attention elsewhere Forgetting
High pain non-conscious generative causal mechanism	Eventually, chronic body-wide pain throughout daily life irrespective of type of movement or whether there is any movement	Eventually, strength of bottom-up signals is not directly related to particular movements or to lack of movements	Eventually, hypervigilance to body-wide pain

**Table 3:** Directions for future research.

Non-Conscious Generative Causal Mechanism (Meta Generative Model)		PGNW Constructs		
		Top-Down	Bottom-Up	Intermediate
		Consistency of Prior Expectations	Strength of Sensory Signals	Attention-Based Modulation
Tendencies	Personality type Hardiness level	How do interactions between them, fascia and body memory affect expectations?	To what extent, if any, do they affect rate and spread of fascia densification?	How do interactions between them, fascia and body memory affect attention?
Powers	Fascia densification Body memory	How does fascia hold and update body memory in the forming of expectations?	How does fascia densification affect intero, noci-, and proprioception?	How does interaction between fascia and body memory affect attention?

## Data Availability

Not applicable.
